# Iron salt supplementation during gestation and gestational diabetes mellitus

**DOI:** 10.11606/s1518-8787.2023057004871

**Published:** 2023-09-29

**Authors:** Vanessa Iribarrem Avena Miranda, Tatiane da Silva Dal Pizzol, Marysabel Pinto Telis Silveira, Sotero Serrate Mengue, Mariângela Freitas da Silveira, Bárbara Heather Lutz, Andréa Dâmaso Bertoldi

**Affiliations:** I Universidade do Extremo Sul Catarinense Programa de Pós-graduação em Saúde Coletiva Criciúma SC Brasil Universidade do Extremo Sul Catarinense. Programa de Pós-graduação em Saúde Coletiva. Criciúma, SC, Brasil; II Universidade Federal do Rio Grande do Sul Faculdade de Farmácia Departamento de Produção e Controle de Medicamentos Porto Alegre RS Brasil Universidade Federal do Rio Grande do Sul. Faculdade de Farmácia. Departamento de Produção e Controle de Medicamentos. Porto Alegre, RS, Brasil; III Universidade Federal de Pelotas Instituto de Biologia Departamento de Fisiologia e Farmacologia Pelotas RS Brasil Universidade Federal de Pelotas. Instituto de Biologia. Departamento de Fisiologia e Farmacologia. Pelotas, RS, Brasil; IV Universidade Federal de Pelotas Departamento de Medicina Social Programa de Pós Graduação em Epidemiologia Pelotas RS Brasil Universidade Federal de Pelotas. Departamento de Medicina Social. Programa de Pós Graduação em Epidemiologia. Pelotas, RS, Brasil

**Keywords:** Pharmacoepidemiology, Drug Utilization, Cohort Studies, Diabetes, Gestational

## Abstract

**OBJETIVE:**

To evaluate the association between the use of iron salts during the first two trimesters of gestation in non-anemic women and the development of gestational diabetes mellitus.

**METHODS:**

The study used maternal data from the 2015 Pelotas Birth Cohort. All non-anemic women at the 24th week of gestation (n = 2,463) were eligible for this study. Gestational diabetes mellitus was self-reported by women. Crude and adjusted logistic regression were performed considering level of significance = 0.05.

**RESULTS:**

Among the women studied, 69.7% were exposed to prophylactic iron supplementation in the first two trimesters of gestation. The prevalence of gestational diabetes mellitus among those exposed was 8.7% (95%CI: 7.4–10.1) and 9.3% (95%CI: 7.4–11.6) among those who were not exposed. Iron supplementation was not associated with increased risk of gestational diabetes mellitus in crude (OR = 0.9; 95%CI: 0,7–1,3) and adjusted analysis (OR = 1.1; 95%CI :0,8–1,6).

**CONCLUSIONS:**

The results suggest that routine iron use in non-anemic pregnant women does not increase the risk of developing gestational diabetes. This evidence supports the existing national and international guidelines, in which prophylactic iron supplementation is recommended for all pregnant women as soon as they initiate antenatal care in order to prevent iron deficiency anemia.

## INTRODUCTION

Gestational diabetes mellitus (GDM) is a temporary condition characterized by hyperglycemia, which occurs due to carbohydrate and glucose intolerance^1^. It begins during pregnancy and usually disappears shortly after delivery^1,2^. Population estimates of hyperglycemia frequency in pregnancy are conflicting, because of the different criteria for GDM diagnosis, with prevalence varying from 1 to 18% of pregnant women in different populations, due to differences between guidelines^2,3^. In Latin America, for example, a GDM diagnosis is the 75 g 2-hour glucose test > 7.8 mmol/L or > 140 mg/dL. In China, > 5.1 mmol/L or > 92 mg/dL sets GDM diagnosis^4^. In Brazil, it is recommended that the GDM diagnosis be established between the 24th and 28th week of gestation, through the oral glucose tolerance test with 75g, when at least one of the following blood glucose values is present: fasting ≥ 92 and < 126 mg/dL; 1 hour ≥ 180 mg/dl; 2 hours ≥ 153 and < 200 mg/dL^2^.

GDM is responsible for many consequences in maternal-fetal health in the short and long term^2^. The most common consequences among children are the increased risk of fetal macrosomia, neonatal hypoglycemia, shoulder dystocia, and hyperinsulinemia at birth. Mothers with GDM are at increased risk of preeclampsia, gestational hypertension, cesarean section, and polyhydramnios. In addition, there is an increased risk of fetal malformations, abortion, neonatal and perinatal mortality, as well as an increased risk of maternal mortality^5,6^. In the long term, the effect of GDM on mothers is the elevated risk for the development of metabolic syndrome and type 2 diabetes^6^. In addition, there is a greater risk of GDM in future pregnancies, which also burdens the public health sector. For the child, the risk of developing childhood obesity and metabolic syndrome almost doubles compared to children born from non-diabetic mothers, and there is also a higher risk of fetal malformation^6^.

Another prevalent problem during pregnancy is iron deficiency anemia, which reaches 42.0% of all pregnant women^7^. In this sense, current guidelines from World Health Organization (WHO) and Brazilian Ministry of Health recommend, in addition to an adequate diet, a prophylactic (routine) iron supplementation for all pregnant women, in order to fulfill the physiological needs for this mineral^8,9^. However, this universal prophylactic supplementation has been questioned, because according to some authors, high iron intake may increase the risk of GDM, especially for women with normal hemoglobin levels in early pregnancy^10,11^, which has been reported as an independent risk factor for DMG^12^.

Findings regarding the association between the use of iron salts and the development of gestational diabetes are new and still inconclusive, therefore, further studies on this topic are necessary. This study aims to evaluate the association between the use of iron salts in non-anemic women in early pregnancy and the development of GDM in a birth Cohort in which mothers were followed up since the antenatal period.

## METHODS

This paper uses data from antenatal and perinatal studies from the 2015 Pelotas’ Birth Cohort (C2015), which was conducted in the city of Pelotas in southern Brazil. The study invited all women living in the urban area of Pelotas who gave birth, including stillbirths, in the city’s five maternity hospitals between January 1 and December 31, 2015.

Mothers were interviewed at the maternity hospital a few hours after delivery and answered a standardized questionnaire. They answered questions about the antenatal period: demographic, socioeconomic, biological and behavioral issues, along with characteristics of pregnancy, delivery, and medication use, including folic acid, iron salts, and other vitamins and minerals. Furthermore, 75% of mothers who participated in the perinatal study were followed by this cohort since the antenatal period. More details on the study can be found in the cohort profile paper^13^.

The analyses in this article were performed with all mothers from the perinatal follow-up who were eligible for this study (n = 2,463) (Figure). Anemic pregnant women before 24th week of gestation (with a record of at least one hemoglobin test < 11 g/dL in their antenatal card) were not included in the analyses.

**Figure:**
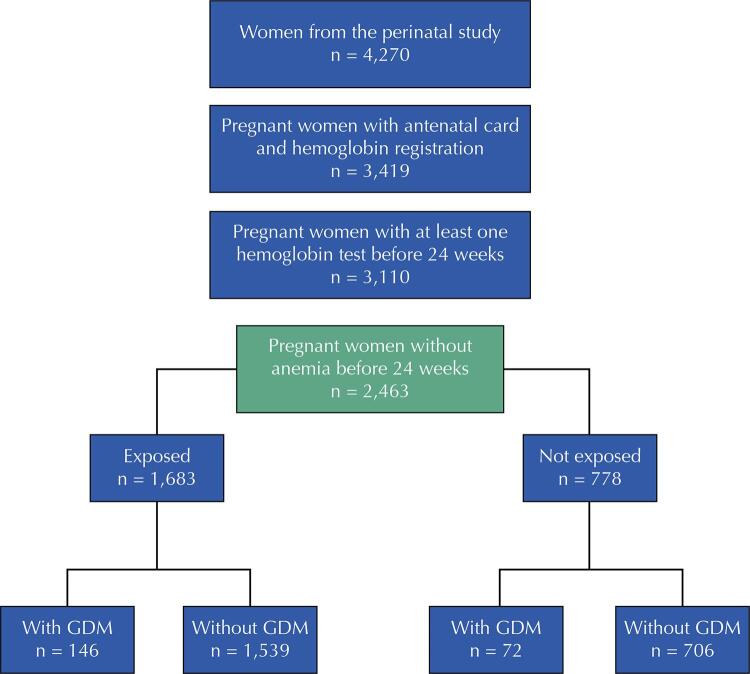
Flowchart of the study population of eligible women. 2015 Pelotas Birth Cohort.

GDM: gestational diabetes mellitus.

For the “gestational diabetes mellitus” outcome, self-reports were considered. The mothers answered the following question: “Did you have diabetes during pregnancy?” followed by another question: “Did you have diabetes before pregnancy?” Pregnant women who had diabetes before pregnancy (n = 44) were excluded. Self-reported information about mother’s knowledge of GDM in the immediate postpartum was validated through a study previously carried out in Pelotas’ maternity, with high specificity (99.0%, 95%CI: 98.1–99.6) and good sensitivity 73.0% (95%CI: 55.9–86.2)^14^.

The variables used in the analysis as possible confounding factors were: maternal age (collected in complete years and categorized as ≤ 19, 20–29, 30–46); ethnicity (self-reported by mothers as white, black or other); parity (total number of deliveries, including stillbirths and current pregnancy; later categorized as 1, 2, 3, or 4 or more); mother’s schooling (number of years of study, later categorized into four groups: 0–4, 5–8, 9–11, and 12 or more years), and family income expressed in local currency and converted into a multiple of minimum wage at the time of the perinatal interview (categorized as ≤ 1, 1.1–3.0, 3.1–6.0, 6.1– 10, and > 10). The ‘minimum wage’ is the measure of the legal minimum monthly salary for formal employees in Brazil.

Family history of diabetes mellitus was reported by mothers at the 24-month follow-up, pre-pregnancy body mass index (BMI) was calculated by dividing pre-pregnancy weight by the square of maternal height, then categorized according to WHO criteria^15^: underweight (< 18.5 kg/m^2^); normal (18.5–24.9 kg/m^2^); overweight (25.0–29.9 kg/m^2^); or obese (≥ 30 kg/m^2^) and smoking in pregnancy was considered as “yes” when the mother reported smoking at least one cigarette a day, for at least 30 days.

Information regarding supplement use was taken from the following questions: *“Have you used or are you currently using any vitamin, calcium, folic acid or iron salts since you became pregnant*?” If the answer was “yes,” the drug names were then questioned and for each reported drug a question about the trimester of use was asked: *“In which trimester of pregnancy did you use this drug?” (*1^st^ trimester, 2^nd^ trimester, 3^rd^ trimester).

From these questions, it was possible to generate the main exposure used in our analyses: “use of prophylactic iron in the first and / or second trimester of pregnancy,” prophylactic iron formulated using only iron compounds or combing them with other active substances, provided that iron was the main compound present. All analyses were performed in Stata software 15.0. Sample description was performed according to exposure and outcome (GDM) using the chi-square test. The association of iron supplements use with GDM development was evaluated by logistic regression.

The regression followed a previously established hierarchical conceptual model, which comprises three levels. The distal level included sociodemographic variables and family history of diabetes; the second level included pre-gestational BMI, parity and smoke; and the proximal level included the use of iron salts. Variables with p < 0.20 were kept in the model to control confounding factors. For all statistical analyses, the significance threshold was set at p < 0.05.

The *Universidade Federal de Pelotas*, School of Physical Education Ethics Committee, approved the study protocol (522.064). All mothers signed an informed consent form before being interviewed.

## RESULTS

Of the 2,463 mothers who participated in this study, about half were aged between 20 and 29 years (48%), most had white skin (75.8%), and more than nine years of schooling (72.1%). The predominant family income was 1.1 to 3 minimum wages (46%), and most mothers (47%) had pre-gestational BMI considered normal by WHO criteria^15^. More than half of women were in their first pregnancy (52.7%), had no family history of gestational diabetes (55.5%) and were non-smokers (86.6%) (Table 1).

**Table 1:** Baseline characteristics of mothers participating in the perinatal cohort study (n = 4,270) and eligible mothers (n = 2,463). 2015 Pelotas Birth Cohort.

Characteristic	Perinatal sample		Eligible mothers	
	
	n (%)	95%CI	n (%)	95%CI
Age				
≤ 19	630 (14.8)	13.7–15.8	282 (11.5)	10.2–12.7
20–9	2,021 (47.3)	45.8–48.8	1,182 (48.0)	46.0–50.0
30–46	1,618 (37.9)	36.4–39.4	998 (40.5)	38.5–42.4
Skin color				
White	3,005 (70.5)	69.1–71.8	1,864 (75.8)	74.1–77.4
Black	680 (16.0)	14.8–17.1	305 (12.4)	11.1–13.7
Others	578 (13.5)	12.5–14.5	291 (11.8)	10.5–13.1
Schooling (years)				
≤ 4	394 (9.3)	8.3–10.1	168 (6.8)	5.8–7.8
5–8	1,098 (25.7)	24.4–27.0	520 (21.1)	19.5–22.7
9–11	1,463 (34.3)	32.8–35.7	894 (36.3)	34.4–38.2
≥ 12	1,314 (30.7)	29.4–32.1	881 (35.8)	33.9–37.6
Family income (minimum wages)^a^				
≤ 1	512 (12.6)	11.6–13.6	222 (9.5)	8.3–10.6
1.1– 3.0	1,906 (47.0)	45.4–48.5	1,077 (46.1)	44.0–48.0
3.1– 6.0	1,077 (26.5)	25.2–27.9	684 (29.2)	27.4–31.1
6.1–10.0	307 (7.5)	6.6–8.2	191 (8.2)	7.0–9.2
> 10.0	257 (6.4)	5.5–7.0	164 (7.0)	5.9–8.1
Pre-pregnancy body mass index (kg/m^2^)				
< 18.5	155 (3.8)	0.3–4.3	62 (2.5)	1.2–3.1
18.5 to < 25.0	1,037 (49.3)	47.8–50.8	1,150 (47.5)	45.5–49.5
25.0 to < 30.0	1,160 (28.1)	26.7–29.4	717 (29.6)	27.8–31.4
≥ 30.0	778 (18.8)	17.6–20.0	490 (20.2)	18.6–21.8
Parity				
1	2,114 (49.6)	48.0–51.0	1,298 (52.7)	50.7–54.6
2	1,315 (30.8)	29.4–32.2	780 (31.7)	29.8–33.5
3	472 (11.0)	10.1–12.0	242 (9.8)	8.6–11.0
≥ 4	367 (8.6)	7.7–9.4	142 (5.8)	4.8–6.6
Family history of GDM^b^				
No	1,707 (55.9)	54.2–57.8	993 (55.5)	53.2–57.8
Yes	1,345 (44.1)	42.2–45.8	795 (44.4)	42.2–46.7
Smoke				
No	3,553 (83.3)	82.1–84.3	2,132 (86.6)	85.2–87.9
Yes	714 (16.7)	15.6–17.8	330 (13.4)	12.1–14.8

95%CI: 95% confidence interval; GDM: gestational diabetes mellitus.

^a^ Family income expressed in local currency and converted into a multiple of minimum wage (R$ 788.00), an amount equivalent to US$ 298.

^b^ Family history of diabetes mellitus gestational. Variable with highest values of missing.

Women eligible for this study did not differ from women participating in the perinatal C2015 study regarding the analyzed characteristics (Table 1).

Of all women in the analyzed sample (n = 2,463), 69.7% were exposed to iron supplementation in the first and / or second trimester of pregnancy. GDM prevalence was higher among pregnant women who were older (12.8%); less educated (13.7%); had a family income between 1.1 and 3.0 minimum / monthly salaries (10.2%); pre-pregnancy BMI ≥ 30 (16.9%); 4 or more children (12.6%) and a family history of diabetes (12.5%) (Table 2).

**Table 2:** Prevalence of gestational diabetes mellitus according to independent variables (n = 2,463). 2015 Pelotas Birth Cohort.

Characteristic	Prevalence of gestational diabetes mellitus			

	n	%	95%CI	p-value^a^
Age				< 0.001
≤ 19	6	2.1	0.9–4.6	
20–29	84	7.1	5.7–8.7	
30–46	128	12.8	10.8–15.1	
Skin color				0.202
White	156	8.4	7.1–9.7	
Black	35	11.4	8.3–15.6	
Others	27	9.2	6.4–13.2	
Schooling (years)				0.004
≤ 4	23	13.7	9.2–19.8	
5–8	37	7.1	5.1–9.6	
9–11	95	10.6	8.7–12.8	
≥ 12	63	7.2	5.6–9.1	
Family income (minimum wages)^b^				0.002
≤ 1	19	8.5	5.5–13.0	
1.1–3.0	110	10.2	8.6–12.2	
3.1–6.0	68	9.9	7.9–12.4	
6.1–10.0	8	4.2	2.1–8.1	
> 10.0	4	2.4	0.9–6.3	
Pre-pregnancy body mass index (kg/m^2^)				< 0.001
< 18.5	1	1.6	0.2–11.1	
18.5 to < 25.0	61	5.3	4.1–6.7	
25.0 to < 30.0	70	9.7	7.7–12.1	
≥ 30.0	83	16.9	13.8–20.5	
Parity				< 0.001
1	83	6.4	5.1–7.8	
2	87	11.2	9.1–13.5	
3	30	12.4	8.7–17.2	
≥ 4	18	12.6	8.0–19.3	
Family history of GDM^c^				< 0.001
No	77	7.8	6.2–9.6	
Yes	100	12.5	10.4–15.1	
Smoke				0.644
No	191	8.9	7.8–10.2	
Yes	27	8.1	5.6–11.6	
Iron supplementation				0.626
No	68	9.3	7.4–11.5	
Yes	146	8.7	7.4–10.1	
Total	214	8.8	7.7–10.0	

95%CI: 95% confidence interval; GDM: gestational diabetes mellitus.

^a^ p-value of chi-square test.

^b^ Family income expressed in local currency and converted into a multiple of minimum wage (R$ 788.00).

^c^ Family history of diabetes mellitus gestational. Variable with highest values of missing.

The prevalence of self-reported GDM in the entire cohort (n = 4,270) was 8.5% (95%CI: 7.6–9.3), and among those eligible for this study it was 8.8% (95%CI : 7.7–10.0), 8.6% (95%CI: 7.3–10.0) being among those who were exposed to iron supplements and 9.2% (95%CI: 8.9–9.2) among those who were not (Table 2).

The use of iron salts in the first or second gestational trimester showed no positive association with GDM both in the crude analysis (OR = 0.9; 95%CI: 0.6–1.3) and in the analysis adjusted for skin color, age, education, family income, pre-gestational BMI, parity, family history of diabetes mellitus, and smoking during pregnancy (OR = 1.1; 95%CI: 0.8–1.5) (Table 3).

**Table 3:** Association between iron salt supplementation and gestational diabetes mellitus among non-anemic women. 2015 Pelotas Birth Cohort.

Iron supplementation	Crude		Adjusted ^a^	
	
	OR (95%CI)	p-value	OR (95%CI)	p-value
Non-anemic pregnant women up to 24^th^ weeks of gestation (n = 2,463)		0.509		0.420
No	Reference (1.0)		Reference (1.0)	
Yes	0.9 (0.7–1.3)		1.1 (0.8–1.6)	
Non-anemic pregnant women up to 12^th^ weeks of gestation (n = 2,559)		0.631		0.405
No	Reference (1.0)		Reference (1.0)	
Yes	1.1 (0.8–1.6)		1.2 (0.8–1.7)	

OR: odds ratio; 95%CI: 95% confidence interval.

^a^ Model adjusted for skin color, age, education, family income in minimum wages, pre-gestational body mass index, parity, smoking during pregnancy, family history of diabetes mellitus.

A subset of analyses was performed for data corresponding to the first trimester of pregnancy (up to 12^th^ weeks). Results were similar to those derived from the main analyses (including the first and/or second trimester (OR_a_ = 1.2; 95%CI: 0.8–1.7) (Table 3).

## DISCUSSION

This study showed that, among non-anemic women, prophylactic use of iron in the first and/or second trimester of pregnancy was not an independent risk factor for the development of GDM.

Published evidence on this subject is controversial. Previous observational studies have suggested that higher stores of iron might be associated with glucose metabolism disorders and an increase in the risk of GDM^16^. This relation is biologically plausible, because iron has a high oxidation-reduction capacity and its free form can catalyze the formation of free radicals, causing cell damage, a process that is also known as oxidative stress. Another suggested mechanism involves the formation of hydroxyl radicals, which can damage the cell membranes of pancreatic ß cells, affecting insulin synthesis and secretion, associated with pregnancy-induced insulin resistance^21,22^. Excessive iron deposition in muscles may also decrease glucose uptake^22^.

However, three randomized controlled trials, performed in hospitals and primary care services in China (n = 1,164)^23^, Finland (n = 2,912)^24^ and the United Arab Emirates (n = 960)^25^, found no significant difference in GDM incidence between control and experimental women. The daily iron dose in the experimental group ranged from 30mg to 60mg administered during the first and second gestational trimesters^24,25^. In the control group, two studies used placebo^24^ and one used a lower dosage of iron and a frequency of use lower than that of the experimental group^25^. It should be noted that one of the randomized clinical trials cited^23^ reported poor adherence to supplementation, which is a reality among pregnant women, mainly due to iron supplementation’s undesirable adverse effects^26^, a fact that may alter the results of this and other researches, underestimating this compound’s real effect.

In our study, no differences were found in GDM prevalence between women exposed and not exposed to the supplement, as in the study of Chan et al.^23^, which did not find increased GDM incidence in the exposed group. The analysis of the study association may be limited due to lack of information regarding dosage, adherence, frequency of use and exposure time. Dosage information would be useful for qualifying the exposure variable (use of iron salts), considering “yes” as a response only for compounds with prophylactic dosage. Knowledge of adherence to iron salt compounds would allow a better selection of women eligible for the study, and exposure time would be useful for estimating dose-response effect.

Regardless of the findings concerning the relationship between the occurrence of GDM and iron use, it is a fact that GDM has been increasing in several countries as a result of population growth, increased maternal age, lack of physical activity and, especially, of the increased prevalence of obesity^2^. Our findings confirm this trend, presenting a prevalence of self-reported gestational diabetes almost three times higher (8.5%; 95%CI: 7.8– 9.6) when compared with another birth cohort study conducted in the year 2004, also in the city of Pelotas, with 4,231 pregnant women, which found a 3.0% self-reported prevalence of gestational diabetes (95%CI: 2.53–3.64)^27^. It is known that there are many divergences between the diagnostic criteria used for the GDM diagnosis, but between 2004 and 2015 there were no changes in the diagnostic test cut-offs in the Brazilian Ministry of Health recommendations^9^.

It should be noted, among the limitations of this study, that many pregnant women had only one or two hemoglobin results in the antenatal card, which means that pregnant women who only had one recorded hemoglobin test with a normal result (non-anemic) were eligible for our study. However, they may have developed anemia later and such information was not registered in the card. On the other hand, the use of information from antenatal records completed by health professionals who followed these pregnant women was a positive aspect and considered indispensable for defining which women were eligible for the study, since these results enabled the identification of non-anemic pregnant women according to the gestational trimester, which guaranteed the temporality of the study association.

The fact that information about the use of iron was only self-reported and that there were no questions about the dose and precise time of treatment can be considered other limitations of this study. Furthermore, the control variable “family history of diabetes” was only collected from the third month of the children’s 24-month follow-up, which resulted in a high number of missing information (27.4%).

Among the positive aspects of the study, we highlight the fact that all pregnant women who had a hospital delivery in the municipality were interviewed within one year, and the small number of refusals, which allows the generalization of the results to other pregnant women under the same conditions of our sample.

## CONCLUSIONS

Our study showed that iron supplementation in non-anamic pregnant women is not related with the development of GDM, which meets current national and international guidelines, whose recommendation is the routine supplementation to all pregnant women as soon as they start antenatal care to prevent iron deficiency anemia.
